# Association of Salivary IGF and IGF/IGFBP-3 Molar Ratio with Cervical Vertebral Maturation Stages from Pre-Adolescent to Post-Adolescent Transition Period—A Cross-Sectional Exploratory Study

**DOI:** 10.3390/ijerph19095172

**Published:** 2022-04-24

**Authors:** Abdullah Almalki

**Affiliations:** Department of Preventive Dental Sciences (Orthodontics), College of Dentistry, Majmaah University, Al-Majmaah 11952, Saudi Arabia; ae.almalki@mu.edu.sa; Tel.: +966-16-404-4319

**Keywords:** orthodontics, insulin-like growth factor, insulin-like growth factor binding protein, skeletal maturity, salivary diagnostics, lateral cephalogram

## Abstract

Background: The relevance of growth determination in orthodontics is driving the search for the most precise and least invasive way of tracking the pubertal growth spurt. Objectives: The aim was to explore whether minimally invasive salivary estimation of biomarkers Insulin-like growth factor (IGF-1) and Insulin-like growth factor binding protein-3 (IGFBP-3) could be used to estimate skeletal maturity with diagnostic accuracy, especially in children and adolescent age groups. Subjects and methods: The cross-sectional study was conducted on 105 participants aged 6–25 years from the out-patient Department of Preventive Dental Science at Majmaah University between the period 2 January 2021 and 12 July 2021. Each subject’s lateral cephalogram radiograph was categorized based on skeletal maturity, and saliva samples were estimated for IGF-1 and IGFBP-3 using the respective ELISA kits. Two-way ANOVA with interaction was applied to examine the main effects due to cervical vertebral maturation staging (CVS), Sex and interaction effect due to CVS, and Sex on study parameters. Karl Pearson’s Product Moment Correlation Coefficient was calculated for finding a significant association between IGF, IGFBP3, and the IGF-1/IGFBP3 molar ratio. Results: Highest mean salivary IGF-1 was observed in the pubertal peak stage, which coincides with cervical vertebral maturity stages 3 and 4 (CVS3 and CVS4) for both males (2.57 ng/mL) and females (1.57 ng/mL) and the lowest mean level of IGF-1 for females (0.85 ng/mL) and males (1.22 ng/mL) was observed during the prepubertal stage. There exists a significant variation in IGF-1 between males and females in the pubertal stage (*p* < 0.01), but the difference is very narrow in the prepubertal and post-pubertal groups (*p* > 0.05). There was no significant interaction effect of different skeletal stages and gender on the IGFBP3 and the IGF-1/IGFBP3 molar ratio (*p* > 0.05), but there exists a significant interaction effect on IGF-1 (*p* < 0.05). Conclusion: Estimation of the IGF-1 and the IGF-1/IGFBP3 molar ratio in saliva, being a non-invasive biological marker, could serve as an adjunctive tool along with radiographic assessment in estimating growth maturity in the adolescence age group. By initiating orthodontic treatment during the mandibular growth peak in adolescence, a positive outcome is ensured in managing skeletal deformities within the craniofacial complex.

## 1. Introduction

Skeletal Age determination is an integral step for clinicians to ascertain the growth status of patients undergoing orthodontic treatment. Timely initiation of orthodontic treatment during the mandibular growth peak ensures a favorable outcome in managing skeletal deformities in the craniofacial complex, as well as it will also alert the clinician of any skeletal growth deficiency or excessive growth, which can jeopardize the eventual outcome of treatment [1].

Characteristic physiologic changes, chronologic age, dental age estimation, and peak height velocity have been previously used to estimate the growing maturity of the individual but several studies have established their clinical irrelevance due to variations concerning gender, ethnicity, and environmental factors [2]. Studies have proved the reliability and predictability of hand–wrist radiographs for the identification of the sequential stages in bone maturation, but the requirement for additional radiographs poses ethical concerns related to radiation hazards [3].

Cervical vertebrae maturation analysis with the help of a lateral cephalogram included staging one to six based on the morphological shape and vertical height of the second, third, and fourth cervical vertebrae. Further, it was modified by Baccetti et al., 2002, into five stages where cervical vertebrae maturation stages 1 and 2 (CVS 1 and CVS 2) were merged into a single stage [4]. Studies have remarked on the complexity and existence of subjective variation while interpreting cephalometric X-rays on an ordinal scale. Further, it lacks accuracy in identifying the span of the growth peak and the decline of the growth period [5]. Henceforth, this aroused the quest for estimating biomarkers related to the growth of high sensitivity and viability, which could assess the growth maturation pattern in a precise way.

Insulin growth factor-1(IGF-1), a polypeptide hormone secreted by the liver, and mediated by growth hormone, has been demonstrated to have a profound role in longitudinal bone growth in the condyle and long bones. Studies have demonstrated the role of IGF-1 in prenatal and postnatal skeletal growth due to the presence of its receptors in mandibular condylar cartilage. Investigators have reported accelerated serum IGF-1 levels from prepubertal till the pubertal peak around 16 years and deceleration in the levels to more than 80% with increasing age during post-puberty [6].

A cross-sectional study by Nayak S et al. in 2014, demonstrated that levels of salivary IGF-I are lowest at the accelerating velocity stage with a steady increase to a peak at the high-velocity stage [7]. The levels were found to decrease mildly at the decelerating velocity stage, but the results exhibited a broad range of standard deviation, which could probably be due to the population’s varied ethnic origin. Therefore, the study participants enrolled for this research were natives from the same region.

Insulin growth factor binding proteins (IGFBPs) function as carriers of IGFs, thereby prolonging the half-life of the hormone and controlling its release and bioavailability. Research implicates that IGFBPs exhibit IGF-1 independent function by their direct association with a variety of extracellular and cell surface proteins, thus mediating bone remodeling function [8]. Studies conducted on children undergoing growth hormone therapy have reported Insulin growth factor binding protein-3 (IGFBP-3) to be a more accurate discriminator of growth hormone-dependent parameters than IGF-1 due to the good reproducibility of IGFBP-3 on repeated testing [9]. The maximum increase in the serum IGF-1/IGFBP-3 molar ratio determines the timing of a pubertal growth spurt, further coinciding with the timing of increased bone formation rate during puberty [10].

Serum estimation for biomarkers being an invasive step has prompted us to explore whether the salivary estimation of IGF-1 and IGFBP-3 could be used to identify prepubertal and pubertal growth spurts in patients. Therefore, this study aimed to estimate levels of IGF-1 and IGFBP-3 levels in whole unstimulated saliva at different cervical maturity stages determined by lateral cephalogram and to identify the correlation of salivary IGF/IGFBP3 molar ratios with different stages of skeletal maturity.

## 2. Materials and Methods

This cross-sectional study was conducted on 105 participants (62 females and 43 males) belonging to the age group of 6–25 years, selected on the basis of convenient sampling from the outpatient Department of Preventive Dental Science between the period 2 January 2021 to 12 July 2021. The study proposal was approved by the institutional ethical committee of Majmaah University, Saudi Arabia (Research Number: MUREC Nov.08/COM-2020/8-2) in accordance with the Helsinki Declaration.

Sample size (*n*) was calculated by the formula

$$\;n=\frac{2S^{2}}{d^{2}}{\left(Z_{\alpha /2}+Z_{1-\beta }\right)}^{2},$$

where *Z_α_*_/2_ = 1.96, *Z*_1−*β*_ = 1.28 are, respectively, the 95% confidence value obtained from the standard normal distribution [10]. At least 30 subjects were required to detect a significant difference in the IGF-1/IGFBP3 molar ratio with an effect size of 0.5 and a standard deviation of 0.6 using a pilot study using 10 respondents. In order to tackle drop-out or non-response, 15% of *n* are added to obtain the final sample size. Hence, the final minimum sample size in each group was 35 each.

In the study, healthy subjects who routinely came for oral examinations and orthodontic consultations were included. Patients who reported any previous history of chronic disease, a disorder related to growth, history of COVID-19, bone disorders, those undergoing active orthodontic treatment, and xerostomia were excluded from the study. Prior to any examination, it was mandatory for all the patients to show their updated version of their immune status with respect to the COVID-19 protocol. Written informed consent form in the Arabic language was obtained before enrolling the patients for the study.

Electronic recordings of the personal details and general examination, such as height, weight, previous medical history, body temperature, previous family, and dental history, were made by a single examiner for ruling out the exclusion criteria. The clinical parameters were recorded for the subjects using a mouth mirror and a community periodontal index probe by a trained investigator. The parameters included simplified oral hygiene index (S-OHI), gingival bleeding index (GBI), community periodontal index (CPI), pocket depth (PD), and clinical attachment loss (CAL) to rule out the periodontal status of the patient [11]. The intra-examiner reliability value was estimated to be 0.81% using kappa statistics. 

**Figure 1:**
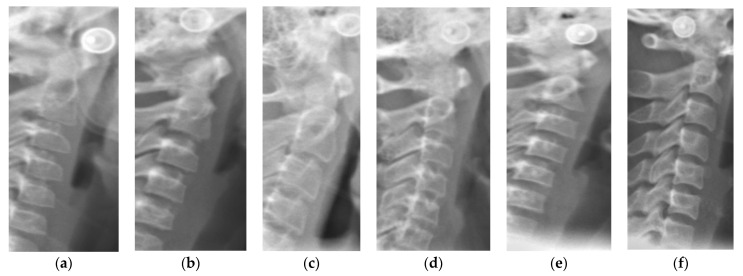
Schematic representation and examples of different cervical maturity stages. CVS: cervical vertebral staging. (**a**) CVS1: Flat lower borders of all the three vertebrae. Trapezoidal body of C3 and C4; (**b**) CVS2: Concavity present at the Lower border of C2. No change in shape of bodies of C3 and C4; (**c**) CVS3: Concavities present at Lower borders of both C2 and C3. C3 and C4 bodies may be either trapezoid or rectangular horizontal in shape; (**d**) CVS4: Concavities present at Lower borders of C2, C3, and C4. C3 and C4 bodies are rectangular horizontal in shape; (**e**) CVS5: Concavities present at Lower borders of C2, C3, and C4. Either one of the bodies of C3 and C4 has square shape others remain rectangular; (**f**) CVS4: Concavities present at the lower borders of C2, C3, and C4. At least one of the bodies of C3 and C4 attained rectangular elongated in shape and the bodies of the other cervical vertebra remain squared.

Lateral cephalograms were taken for all the recruited subjects with radiographic exposure at 80 kV, 9 mA for 1.25 s as a part of the routine orthodontic diagnostic and treatment protocol, thus avoiding the ethical concerns of additional radiation exposure as in Handwrist radiographs. All the radiographs were taken using the same digital X-ray equipment, Kodak 8000 panoramic, and cephalometric system. Radiographs were evaluated using Apteryx Imaging software. Two examiners, who were blinded to the patient’s personal and clinical details, interpreted the radiographs and categorized them into 3 groups depending on the cervical vertebral maturation staging (CVS1-CVS6) as proposed by Baccetti et al. [12] (Figure 1). Inter-examiner reliability and intra-examiner reliability were estimated using Kappa statistics. The groups representing different skeletal maturity stages are as follows:

Group 1: CVS1 and CVS2 pre-pubertal stage.
Group 2: CVS3 and CVS4 pubertal stage.
Group 3: CVS5 and CVS6 post-pubertal stage.

All subjects were advised to report during the time 10 A.M. to 12 A.M. to standardize the diurnal variation. They were seated upright in a comfortable position and advised to rinse the mouth thoroughly before collecting the saliva on the floor of the mouth. Then, 5 mL of unstimulated saliva was collected in a graduated epindroff tube by the spitting method [13]. After collection, all tubes were labeled by a single examiner and the samples were stored at −20 °C until further analysis.

The levels of IGF-1 and IGFBP-3 in all samples were assessed using Enzyme-Linked Immunosorbent Assay kits (ELISA) within 4 months. The stored saliva was thawed and centrifuged (Kenley, London, UK, rotor radius 7 cm) at 3500 RPM for 10 min to remove proteins; the clear supernatant was collected to estimate Insulin-Like Growth Factor 1 (IGF-1); Insulin-Like Growth Factor Binding Protein 3 (IGFBP3). ELISA kits for IGF1 and IGFBP3 (Cloud-Clone Corp, Katy, TX, USA) were used as per the manufacturer’s instructions.

The data were summarized as frequency, percentage, mean, and SD. Two-way ANOVA was used to relate the main effects and the interaction effects of the different groups, and gender on IGF-1, IGFBP3, and the IGF-1/IGFBP3 molar ratio after assessing the basic assumptions of linear regression models such as heteroscedasticity, multicollinearity, and normality of the residual variance. In this study, two main effects considered were, the effect of group (1, 2, and 3) and gender (Male, Female); and the interaction effect of gender and skeletal maturity over IGF1, IGFBP3, and the IGF-1/IGFBP3 molar ratio. The strength of association of main effects and interaction effects was assessed with the help of Partial Eta Squared. According to Cohen, an effect is considered small, medium, or large if partial Eta squared are 0.01, 0.06, and 0.14, respectively [14]. 

Two-way ANOVA with interaction was applied to examine the main effects due to cervical vertebral maturation staging (CVS), Sex and interaction effect due to CVS, and Sex on study parameters. Scheffe’s post hoc pairwise comparisons were also made using Bonferroni adjustments in observed *p* values. Karl Pearson’s Product Moment Correlation Coefficient was calculated for finding a significant association between IGF-1, IGFBP3, and IGF-1/IGFBP3. A 95% confidence interval for the correlation coefficient (L0.05 and U0.05) was also calculated, and the significance of the observed correlation was tested using a t-test. A *p*-value less than 0.05 was considered to be statistically significant. All analyses were carried out with the help of SPSS v.23 for WINDOWS.

## 3. Results

A total of 105 subjects were included in the study with 35 subjects recruited in each group. Table 1, shows that a majority of male patients belong to the 14–20 years of age group (37.2%) while the majority of female patients belong to the less than 14 and greater than 14 years of age group (41.9%). The total sample majority of patients belong to the less than 20 years of age group (38.1%). Most of the patients in the sample belonged to rural areas (67.6%). The frequency shows that a majority of patients performed brushing once a day (60.0%). Most of the male patients had occasional previous dental visits (34.9%), while it was observed that most female patients had a dental checkup at least once a year (46.8%). The majority of patients reported a good oral hygiene status, which was assessed using OHI-S (61.0%). Most of the patients demonstrated healthy periodontium, which was clinically assessed by a GBI score of less than 10% of the sites (56.2%), probing pocket depth of 1–3 mm (80.0%), CAL score of 0 (87.6%), and CPI score of 0–1 (55.2%). About 10.5% demonstrated a CPI score of three, indicating periodontal disease; 6.7% of the participants had mild CAL, 3.8% moderate, and 1.9% of participants demonstrated severe attachment loss.

From the two-way ANOVA analysis given in Table 2, it can be inferred that there exists no significant interaction effect of different skeletal stages and gender on IGFBP3 and the IGF-1/IGFBP3 molar ratio (*p* > 0.05), but there exists a significant interaction effect on IGF-1 (*p* < 0.05). It may also be noted that there is no significant main effect due to gender on the IGF-1/IGFBP3 molar ratio (*p* > 0.05).

**Figure 2:**
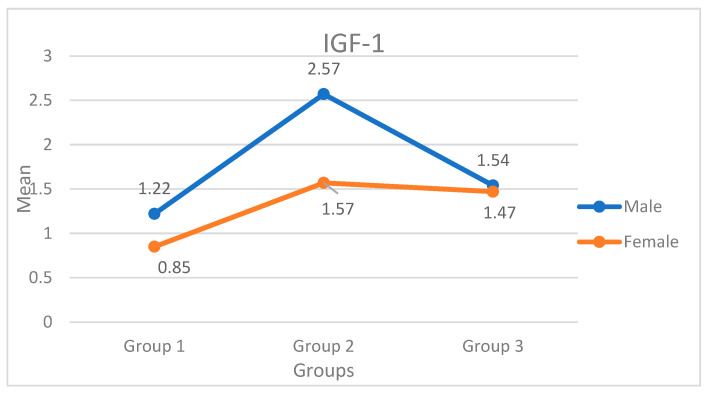
Graphical representation showing IGF-1 levels based on groups and gender.

From Table 3, Figure 2, the least mean level of IGF-1 for females and males is reported in group 1 (0.85 and 1.22), while the highest mean IGF-1 is reported in group 2 for both males (2.57) and females (1.57). There exists a significant variation in IGF-1 between males and females in group 2 (*p* < 0.01), but the difference is very narrow in group 1 and group 3 (*p* > 0.05).

There exist significant gender differences in the means of IGFBP3 (*p* < 0.01). The overall mean for males (3.92) is significantly higher than for females (3.49). The overall mean from group 2 (3.79) is significantly higher as compared to group 3 (3.68) and group 1 (3.54). In both the intergroup and intragroup (p > 0.05), there is no statistically significant gender difference in the IGF-1/IGFBP3 molar ratio. In both the intergroup and intragroup, there is no statistically significant gender difference in the IGF-1/IGFBP3 molar ratio (*p* > 0.05), but the overall mean is significantly higher for group 2 (1.80) compared to group 3 (1.54) and group 1 (1.07). Scheffe’s post hoc test shows that the variation was not significant between group 2 and group 3 (*p* > 0.05) (Table 2). 

Table 4 shows that the correlation between IGF-1 and IGFBP3 is moderately positive (0.360, 0.194 to 0.526), and the results are found to be statistically significant (*p* < 0.01). The correlation between IGF-1 and the IGF-1/IGFBP3 molar ratio is strongly positive (0.942, 0.920 to 0.964) and significant (*p* < 0.01), but no significant relationship exists between IGFBP3 and the IGF-1/IGFBP3 ratio (*p* > 0.05). 

## 4. Discussion

Assessment of skeletal growth acceleration in dentofacial orthopedics serves a predominant role in optimizing timings for functional orthodontic interventions to treat skeletal imbalances. Both females and males demonstrated the lowest mean levels of IGF-1 in the prepubertal group, while the highest mean level of IGF-1 was found in the pubertal group for both genders. Significant gender differences were observed in the salivary levels of IGF-1 and IGFBP3. Overall, males demonstrated significantly higher mean levels of IGF-1 and IGFBP3 than females. Cervical vertebral staging suggested by Baccetti et al. has gained much popularity due to its ease of analysis and high reproducibility among various researchers. According to Baccetti et al., cervical vertebral maturity staging 1 and 2 (CVS1 and CVS2) take place before the mandibular growth peak; the peak growth occurs between the CVS3 and CVS4 stages, while the CVS5 and CVS6 stages occur after the peak [12]. Studies also observed that skeletal maturity occurs even after the growth spurts, and hence it is important to focus on the span of mandibular growth and its decline to plan various orthodontic treatment modalities [15].

IGF-1 is considered as a mediator of growth hormone function, involved in the growth of almost every organ, and plays a major role in postnatal growth and precisely in the process of longitudinal bone growth [16]. Furthermore, IGF-1 levels do not fluctuate through the day like GH levels, making IGF-1 a useful diagnostic tool for determining GH status. Henceforth, assessment of growth-specific biomarkers such as IGF-1 could enhance the predictability and accuracy of estimating growth in craniofacial orthopedics. This study focused on analyzing salivary levels of IGF-1, IGFBP-3, and IGF-1/IGFBP-3 molar ratios on 105 subjects belonging to age groups 5–25 years. They were divided into three groups according to different skeletal maturity stages as proposed by Bacetti using a lateral cephalogram, which was obtained as a pretreatment diagnostic record [12].

Results of the study demonstrated the highest mean salivary IGF-1 in the pubertal peak stage for both males (2.57 ng/mL) and females (1.57 ng/mL), and the lowest mean level of IGF-1 for females (0.85 ng/mL) and males (1.22 ng/mL) was observed during the prepubertal stage that coincides with CS1 and CS2. Significant variation in IGF-1 between males and females was noted during the pubertal stage (*p* < 0.01), but variation between the intragroup was not significant for the prepubertal and post-pubertal stages.

The findings of this study are in accordance with Gupta et al. where serum IGF-1 levels were presented according to the descending order of skeletal maturity stages CVS3 > CVS4 > CVS5 > CVS2 > CVS6 > CVS1 in females and CVS4 > CVS5 > CVS3 > CVS2 > CVS6 > CVS1 in males, where the highest levels were observed during the pubertal stage and the least were observed during the prepubertal stage [17]. Baccetti stated that the ideal stage to begin functional orthopedics for class II malocclusion is CS3, even if peak mandibular growth speed differs in chronological ages [12]. Many studies demonstrated supporting evidence for peak IGF-1 values observed during the pubertal stage. Moreover, peak levels correlated with CS3, and a gradual decline in the IGF-1 levels were observed in CS5 and CS6 [8]. A longitudinal study conducted by Masood et al. 2012, monitored periodic mandibular growth for a year and found that increased levels of serum IGF-1 of more than 250 ng/mL demonstrated mandibular growth of 5.5 mm [18].

The significant variation in the IGF-1 levels with genders in the pubertal peak is consistent with the observation by Ishaq et al., 2012, who reported a significant difference in the levels of IGF-1 between males and females in stage CS5 [19]. Varying reference ranges in the serum IGF-1 levels in studies could be due to discordance attributed to ethnicity, variation in the method of analysis, and environmental and genetic factors. In this study, salivary IGF-1 levels were not estimated stage-wise because of the unavailability of a sufficient sample size for each stage. Therefore, the cervical maturity stages were grouped according to the various periods in a growth spurt. The observations in this study contradict the results reported by Brabent et al., 2003, where females demonstrated greater values than males during adolescence. Females reported earlier and shorter growth spurts compared to males, who were found to be having delayed and longer growth spans [20]. 

Jain S et al. in 2013 demonstrated that serum IGF-1 levels of cervical staging CVS3, CVS4, and CVS5 functional orthopedic treatment could be initiated if IGF-1 values were more than >310 ng/mL. However, the study was conducted on IGF-1 levels in blood serum of male subjects only, leaving doubts about the quantification of IGF-1 levels in women [21]. Quantification of Salivary levels of IGF-1 in this study for the corresponding stages ranged between 1.47 and 2.57 ng/mL. These findings support the reports by Yasuda Y stating that the expression in the saliva is less than 1% in contrast to levels analyzed in serum [22]. Salivary diagnostics have the advantage of being non-invasive compared to serum, but scarce literature exists correlating salivary IGF-1 levels with skeletal maturity. Despite decreased expression, results of this study showed that salivary IGF-1 followed the same variation pattern as in serum and, hence, could be used as a potential non-invasive diagnostic marker, but further longitudinal studies are warranted to establish reference ranges concerning facial growth assessments. 

A recent study by Carelli J et al., 2021, demonstrated that only a moderate correlation existed between serum IGF-1 levels and radiographic skeletal maturity indicators, suggesting the possible role of IGF-1 even after the growth spurt in the mandibular growth span [15]. Probably, this could be the reason for a continued increase in salivary IGF-1 in females even after puberty observed in this study.

Further, it was observed that the values in the post-pubertal group remained higher than in the pre-pubertal group, which supports the findings by Phogat R et al. in 2018; the levels in the growth maturation and completion stages demonstrated higher values than in the initiation and acceleration stage [23]. The continued increase in the IGF-1 levels indicates variable growth patterns and the existence of residual mandibular growth even after CVS5. These findings are in contradiction with the assessment performed by Masood et al. 2008, which could probably be due to the difference in the method of estimating the biomarker or the difference in ethnicity among the heterogeneous population [3]. The biological reason for sexual dimorphism observed in this study could be due to the contribution of the sex hormones in modulating the levels of IGF-1 secretion from the liver. The indirect effect on the growth hormone by estrogen brings about a decrease in IGF-1 feedback inhibition, whereas testosterone has a direct effect on the growth hormone [24].

IGFBP-3 acts as a carrier protein for IGF-1, hence decreasing its bioavailability in circulation. Growth hormone-mediated release of IGF-1 and IGFBP-3 in circulation is controlled by various factors. The relationship between IGF-1 and IGFBP-3 demonstrates an opposite biological action where IGFBP-3 plays a pivotal role in controlling the mitogenic capacity of IGF-1, further exhibiting its role in bone remodeling [10]. Therefore, in this study, IGFBP-3 and the IGF-1/IGFBP-3 molar ratios were evaluated to analyze the actual effective level of IGF-1 in circulation. To date, no literature has been published relating to salivary IGFBP-3 levels and the IGF-1/IGFBP3 ratio with different radiographic skeletal maturity stages.

The results of the study demonstrated a positive interaction effect between different skeletal maturity groups and salivary IGFBP-3 levels. Mean Salivary IGFBP3 showed variation between the genders, (*p* < 0.01) with males demonstrating higher levels than females. The highest values are shown to be reflected in the pubertal group followed by the post-pubertal, and the least are in the prepubertal group. These findings were consistent with the observations by Lofqvist C et al. 2005, where they estimated the reference values for serum IGFBP3 and the IGF-1/IGFBP3 ratio from the childhood to adolescence period. They inferred that serum IGFBP3 was least in early childhood and was found to increase progressively till the highest peak levels were observed during the sexual maturation period, when gonadal steroids are elevated between 12 and 14 years. The serum IGFBP3 levels demonstrated a negative association from the late mid-pubertal stage and the levels were maintained at a constant rate in late puberty [25].

The two-way ANOVA test demonstrated no significant interaction on the effect of salivary levels of IGFBP3 and the IGF-1/IGFBP3 ratio with different skeletal age groups and gender, but there exists a significant interaction effect on IGF-1(*p* < 0.05). The study results did not demonstrate any significant main effect due to gender on the IGF-1/IGFBP3 ratio. These findings were not in agreement with the observation by Lofquist C et al., where higher levels of the IGF-1/IGFBP3 ratio were observed in females during the mid-pubertal period compared to males [25]. This further supports the fact that the peak height velocity observed earlier in girls compared to boys can be due to the earlier rise in IGF-1 levels in girls than in boys. Since gender differences were observed in the study, reference ranges were estimated for serum IGFBP-3 and the IGF-1/IGFBP-3 molar ratio for pubertal stages for both boys and girls. This would be a favorable indicator for orthopedic growth maturity assessment.

An extensive study conducted by Kanbur NÖ et al. compared pubertal development with Tanner’s criteria and concluded that levels of IGF-1 and the IGF/IGFBP3 ratios in blood serum correlated with skeletal age assessed by estimating osteocalcin and bone alkaline phosphate levels during pubertal development. A significant correlation was found between serum IGF-1 and IGFBP-3 levels with osteocalcin and bone-specific alkaline phosphatase levels in boys, but not in girls [26]. It was seen that the correlation between IGF-1 and IGFBP3 is moderately positive (0.360, 0.194 to 0.526) and significant (*p* < 0.01). The correlation between IGF-1 and IGF-1/IGFBP3 is very highly positive (0.942, 0.920 to 0.964) and significant (*p* < 0.01); however, no significant relationship exists between IGFBP3 and IGF-1/IGFBP3 (*p* > 0.05).

However certain limitations of this study exist that resulted in inconsistent gender differences, which could be due to unmatched gender samples belonging to each stage, ethnic variation, cross-sectional study design, periodontal disease, and minimal expression of growth markers in saliva. Further longitudinal studies are warranted with respect to the same after adjustment of the confounding factors, namely, ethnicity, hormonal variation, and nutritional deficiency, which can act as systemic conditions affecting the tissue response to inflammation as well as the expression of the biomarkers in saliva.

## 5. Conclusions

As a non-invasive biological marker, the IGF-1 and the IGF-1/IGFBP3 ratio in saliva could prove to be a valuable tool in conjunction with other biological indicators for the assessment of orthodontic growth maturity; however, further longitudinal studies are warranted to assess reference ranges concerning skeletal growth.

## Figures and Tables

**Table 1:** Frequency and percentage distribution of patients based on gender according to various study parameters.

Variable	Class	Male *N* (%)	Female *N* (%)	Total *N* (%)
Age (years)	Less than 14	14 (32.6)	26 (41.9)	40 (38.1)
	14 to 20	16 (37.2)	10 (16.2)	26 (25.8)
	Greater than 20	13 (30.2)	26 (41.9)	39 (37.1)
Location	Urban	9 (20.9)	25 (40.3)	34 (32.4)
	Rural	34 (79.1)	37 (59.7)	71 (67.6)
Family history of periodontal disease	Yes	8 (18.6)	12 (19.4)	20 (19.0)
	No	35 (81.4)	50 (80.6)	85 (81.0)
Frequency of brushing	Once	29 (67.4)	34 (54.8)	63 (60.0)
	Twice	8 (18.6)	28 (45.2)	36 (34.3)
	occasionally	6 (14.0)	0 (0.0)	6 (5.7)
Previous dental visits	Once a year	14 (32.6)	29 (46.8)	43 (41.0)
	Twice a year	6 (14.0)	16 (25.8)	22 (21.0)
	Occasionally	15 (34.9)	13 (21.0)	28 (26.7)
	Never	8 (18.5)	4 (6.4)	12 (11.3)
Simplified Oral hygiene index (OHI-S)	Good	25 (58.1)	39 (62.9)	64 (61.0)
	Fair	11 (26.6)	20 (32.3)	31 (29.5)
	Poor	7 (16.3)	3 (4.8)	10 (9.5)
Bleeding on probing % of sites	No BOP	22 (51.2)	37 (59.7)	59 (56.2)
	<10%	4 (9.3)	6 (9.7)	10 (9.5)
	10–30%	5 (11.6)	9 (14.5)	14 (13.3)
	>30%	12 (27.9)	10 (16.1)	22 (21.0)
Probing pocket depth	1–3 mm	33 (76.7)	51 (82.3)	84 (80.0)
	4–5 mm	10 (23.3)	9 (14.5)	19 (18.1)
	>5 mm	0 (0.0)	2 (3.2)	2 (1.9)
Community periodontal index (CPI)	0	22 (51.2)	36 (58.1)	58 (55.2)
	1	6 (14.0)	6 (9.7)	12 (11.4)
	2	10 (23.2)	12 (19.4)	22 (21.0)
	3	5( 11.6)	6 (9.7)	11 (10.5)
	4	0 (0.0)	2 (3.1)	2 (1.9)
Clinical attachment loss (CAL)	No CAL	38 (88.4)	54 (87.1)	92 (87.6)
	1–2 mm	5 (11.6)	2 (3.2)	7 (6.7)
	3–4 mm	0 (0.0)	4 (6.5)	4 (3.8)
	5 mm and more	0 (0.0)	2 (3.2)	2 (1.9)
Groups	Group 1	18 (41.9)	17 (27.4)	35 (33.3)
	Group 2	9 (20.9)	26 (41.9)	35 (33.3)
	Group 3	16 (37.2)	19 (30.7)	35 (33.4)

*N*: number, %: percentage.

**Table 2:** Two-way ANOVA relating to main effects and interaction effects of different groups and gender on IGF-1, IGFBP3, and the IGF-1/IGFBP3 molar ratio.

Parameter	Effects	F	*p*	Partial Eta Square
IGF-1	Group	17.31	<0.0005 **	0.26
	gender	11.74	0.001 **	0.11
	group * gender	3.56	0.032 *	0.07
IGFBP3	Group	4.32	0.016 *	0.08
	gender	23.37	<0.0005 **	0.19
	group * gender	0.52	0.597 NS	0.01
IGF-1/IGFBP3 Molar Ratio	Group	12.91	<0.0005 **	0.21
	gender	3.33	0.071 NS	0.03
	group * gender	2.65	0.076 NS	0.05

**: Highly significant (*p* < 0.01), *: Significant ( *p* < 0.05), NS: Not Significant, IGF-1: Insulin growth factor-1, IGFBP-3: Insulin growth factor binding protein-3.

**Table 3:** Mean (SD) of IGF-1, IGFBP3, and the IGF-1/IGFBP3 ratio based on groups and gender.

IGF-1			IGFBP3		IGF1 /IGFBP3 Molar Ratio	
Group	Male	Female	Male	Female	Male	Female
1	1.22 (0.67)	0.85 (0.77)	3.84 (0.20)	3.21 (0.60)	1.18 (0.66)	0.96 (0.84)
2	2.57 (0.33)	1.57 (0.49)	4.15 (0.30)	3.67 (0.61)	2.31 (0.31)	1.62 (0.52)
3	1.54 (0.88)	1.47 (0.77)	3.89 (0.34)	3.50 (0.62)	1.47 (0.83)	1.60 (0.77)

IGF-1: insulin growth factor-1, IGFBP-3: insulin growth factor binding protein-3.

**Table 4:** Correlation(r) among IGF-1, IGFBP3, and the IGF-1/IGFBP3 molar ratio.

Correlation	N	r	SE	L0.05	U0.05	*p*
IGF-1 and IGFBP3	105	0.360	0.085	0.194	0.526	<0.0005 **
IGF-1 and IGF-1/IGFBP3 Molar Ratio	105	0.942	0.011	0.920	0.964	<0.0005 **
IDFBP3 and IGF-1/IGFBP3 Molar Ratio	105	0.069	0.097	−0.121	0.259	0.482 NS

**: Highly significant (*p* < 0.01), NS: Not Significant. r: correlation coefficient, SE: standard error from correlation coefficient, IGF-1: insulin growth factor-1, IGFBP-3: insulin growth factor binding protein-3.

## Data Availability

Data supporting reported results can be presented on request.
